# Analysis of diastolic left ventricular wall shear stress in normal people of different age groups

**DOI:** 10.3389/fcvm.2022.953384

**Published:** 2022-09-23

**Authors:** Liping Dong, Hairu Li, Xiangli Xu, Min Ren, Weidong Yu, Wenkun Bai, Di Sun, Jiawei Tian

**Affiliations:** ^1^Department of Ultrasound Medicine, The Second Affiliated Hospital of Harbin Medical University, Harbin Medical University, Harbin, China; ^2^Department of Ultrasound Medicine, The Second Hospital of Harbin, Harbin, China; ^3^Department of Ultrasound Medicine, Shanghai First Maternity and Infant Hospital, Tongji University School of Medicine, Shanghai, China; ^4^Department of Ultrasound Medicine, Shanghai Sixth People’s Hospital, Shanghai Jiao Tong University School of Medicine, Shanghai, China

**Keywords:** echocardiography, vector flow mapping, wall shear stress, hemodynamics, left ventricle (LV)

## Abstract

**Background:**

Diastolic wall shear stress (WSS), assessed by using vector flow mapping (VFM), is the result of the interaction between the blood flow and the ventricular wall. This study aimed to evaluate the trend of left ventricular (LV) WSS in normal subjects.

**Methods and results:**

A total of 371 healthy volunteers were recruited and divided into four age groups (group I: 18–30 years; group II: 31–43 years; group III: 44–56 years; group IV: 57–70 years). LV WSS of different age groups was measured at each diastolic phase (P1: isovolumic diastolic period, P2: rapid filling period, P3: slow filling period, and P4:atrial contraction period) to evaluate the change trend of LV WSS. In each age group, LV WSS coincided with a trend of increasing-decreasing-increasing during P1–P4 (*P* < 0.05). Besides, among groups I, II, III, and IV, WSS of anterolateral, inferoseptal, and anteroseptal in P1 and WSS of inferolateral, inferoseptal, and anteroseptal in P4 all showed an increasing trend with age (*P* < 0.05). Regarding sex differences, women had greater diastolic WSS compared to men (*P* < 0.05).

**Conclusion:**

LV WSS showed a regular variation and had specific age- and sex-related patterns in different diastolic phases.

## Introduction

In the past few years, increasing attention has been dedicated to the intraventricular flow pattern. The flow pattern is the consequence of the heart chiral geometry and the interaction between the filling jet with the wall and valves of the ventricle, revealing the exceptional adaptability of the cardiovascular system for maintaining relatively constant blood circulation under high workloads (1, 2). Abnormal flow pattern within the ventricular chamber is related to many sorts of cardiac dysfunction, such as myocardial ischemia (3), cardiomyopathy (4), and thrombosis (5). Therefore, the flow pattern may offer a novel index of cardiac dysfunction (6).

Vector flow mapping (VFM), used in combination with color Doppler and two-dimensional speckle tracking technique, is a novel echocardiographic technology that can visualize the intraventricular flow pattern (7). Blood flow visualization studies provide clues to physiological and pathophysiological mechanisms by which abnormal flow pattern increases cardiac workload and deteriorates ventricular function (8, 9). Energy loss (EL), circle, and wall shear stress (WSS), derived from the intraventricular flow vector field, are parameters reflecting the spatial dispersion of the intraventricular flow pattern. Most previous studies on VFM have focused on EL and circle (3, 10), but recently, WSS has been reported to be a new quantification parameter (11) because it might indicate the underlying association between fluid mechanics and cardiovascular diseases (CVDs) (12). WSS plays a key role in regulating endocardial cells and triggers a series of biological signal transductions, which, in turn, regulate gene expression and the function of vascular wall cells (13, 14). For example, during the embryonic stage, WSS can influence the development of the original heart by adjusting the endocardial cells (15, 16). Low shear stress can promote vascular endothelial cell proliferation, while high shear stress can inhibit it (17).

Although WSS has been reported useful in previous studies, little has been known about its change in healthy adults. This study aimed to explore WSS variation in the left ventricle (LV) during the diastolic period and analyze the differences stratified by age and sex.

## Materials and methods

### Study population

A total of 371 healthy volunteers (60.6% women), with a mean (standard deviation, SD) age of 43 years (17), were recruited from the physical examination center of the Second Affiliated Hospital of Harbin Medical University from August 2018 to March 2019. Afterward, they were separated into four groups according to their age quartile segmentation (group I: 18–30 years; group II: 31–43 years; group III: 44–56 years; group IV: 57–70 years). Age quartile segmentation refers to calculating the age quartile of the subjects and then dividing the subjects into four subgroups according to the quartile. The inclusion criteria comprised no abnormalities at clinical presentation, no history of any comorbidity, and sinus rhythm on electrocardiogram (ECG). According to the 2019 guidelines of the American Society of Echocardiography, the subjects also met the following criteria: Ave E/e′ of < 14, mitral septal e′ of < 7cm/s or mitral lateral e′ of < 10 cm/s, and LAVI of < 34 ml/m^2^. The study conformed to the principles outlined in the Declaration of Helsinki and Good Clinical Practice guidelines and was approved by the ethical committee of the Second Affiliated Hospital of Harbin Medical University (reference no. KY2019-222).

### Image acquisition

**FIGURE 1:**
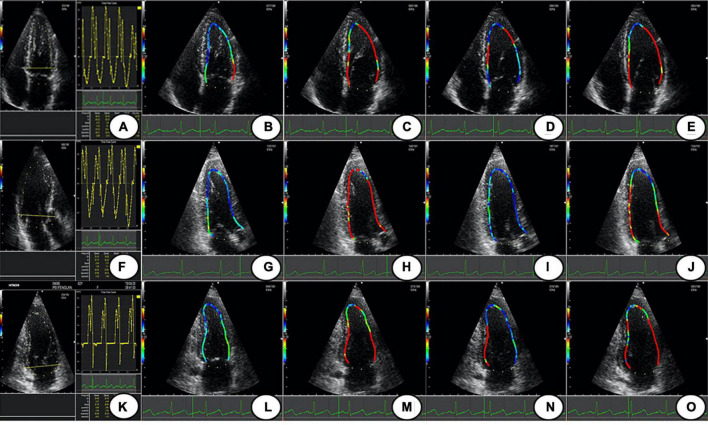
The LV WSS of Apical 4 C, 3C, and 2C; **(A,F,K)** are the time-flow curves of Apical 4C, 3C, and 2C. **(B–E)** The LV WSS of Apical 4C in P1–P4; **(G–J)** the LV WSS of Apical 3C in P1–P4; **(L–O)** the LV WSS of Apical 2C in P1–P4. Apical 4C, apical 4-chamber; Apical 3C, apical 3-chamber; Apical 2C, apical 2-chamber; P1, isovolumic diastolic period; P2, rapid filling period; P3, slow filling period; P4, atrial contraction period; LV WSS, left ventricular wall shear stress.

The study was performed using Hitachi Aloka LISENDO 880 ultrasound system (Hitachi-Aloka Medical, Ltd., Tokyo, Japan) with the phased array single crystal probe (probe frequency: 1.0–5.0 MHz, frame rate: 66–78 Hz). All subjects were connected to a 12-lead ECG and examined in the left lateral decubitus position. Conventional and tissue Doppler transthoracic echocardiography (TTE) was performed by experienced sonographers and reviewed by senior physicians. In two-dimensional (2D) echocardiographic assessment, the left ventricular end-diastolic diameter (LVEDd), left ventricular end-systolic diameter (LVESd), end-diastolic septal thickness (IVST), left ventricular posterior wall thickness (LVPWT), and left atrial volume index (LAVI) were measured. In pulsed wave (PW)/tissue Doppler imaging (TDI) evaluation, the early diastolic peak velocity (E) and late diastolic peak velocity (A) of the mitral valve were measured in the apical four-chamber view. Then, the E/A ratio was calculated. The early diastolic peak velocity of the mitral annulus (medial e′ and lateral e′) was also measured for further calculation of medial and lateral E/e′. The left ventricular ejection fraction (LVEF) was generated by the Biplane Simpson method. Regarding the VFM mode, the 2D gain was adjusted to optimize the visualization of the endocardium, mitral valve, and aortic valve. The size of the sampling frame was also adjusted to completely envelop the LV, while the pulse repetition frequencies (PRFs = 4 kHz) for color Doppler flow imaging (CDFI) were set high enough; hence, the flow filled the LV without aliasing and blood flow spilling. The 2D and CDFI images of the apical 4-chamber (Apical 4C), 3-chamber (Apical 3C), and 2-chamber (Apical 2C) views were recorded (Figure 1).

### Data analysis

**FIGURE 2:**
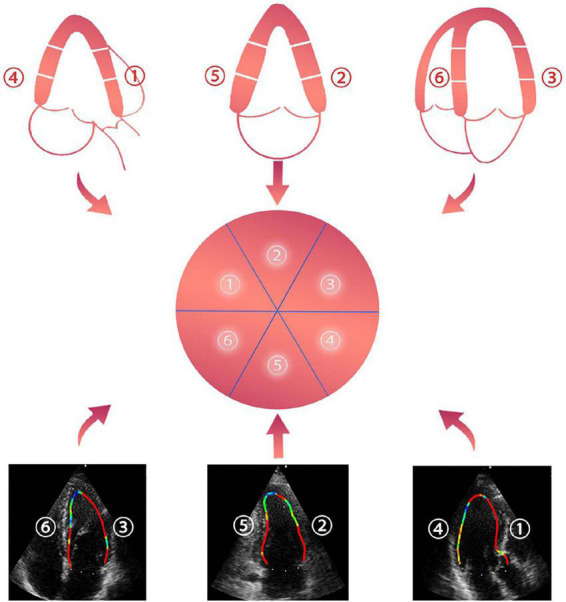
➀ anteroseptal view, ➁ anterior view, ➂ anterolateral view, ➃ inferolateral view, ➄ inferior view, and ➅ inferoseptal view.

The acquired images were imported into the DAS-RS1 workstation for offline analysis. First, the endocardial border was traced manually at the clearest frame, and the software automatically traced the remaining frames. The user was allowed to check and edit the image frame by frame. The traced images of the Apical 4C, 3C, and 2C were processed on the offline VFM workstation. As a result, we obtained the rate data of LV WSS. The LV wall was divided into six segments: anterior, anterolateral, inferolateral, inferior, inferoseptal, and anteroseptal (Figure 2). Similarly, LV WSS was divided into six parts. The diastolic period was defined as the first frame after aortic valve closure to the first frame after mitral valve closure. Then, the diastolic period was divided into four phases based on the ECG, time-flow curve, and two-dimensional cardiac valvular opening and closing, including an isovolumic diastolic period (P1), rapid filling period (P2), slow filling period (P3), and atrial contraction period (P4).

**FIGURE 3:**
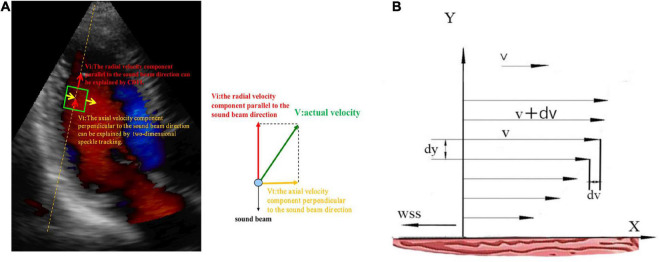
**(A)** The blood flow vector of the left ventricle in the apical view. Vi, the blood flow vector parallel to the sound beam; Vt, the blood flow vector perpendicular to the sound beam; V, the actual blood flow vector. **(B)** *X*-axis, left ventricular wall; v, the flow velocity of a certain blood flow layer; v + dv, the flow velocity of adjacent blood flow layer; dv, the velocity difference; dy, the vertical distance between two flow layers; WSS, the direction of WSS is opposite to the direction of blood flow.

Since the VFM technique is a combination of CDFI and 2D speckle tracking, the radial blood flow velocity, which is parallel to the sound beam direction, can be measured by the CDFI, and the 2D speckle tracking can be used to measure the axial blood flow velocity, which is perpendicular to the sound beam direction. Then, we could acquire both the radial and axial flow velocities, allowing the continuity equation to calculate the actual intraventricular flow (Figure 3A) (18). The Newton inner friction equation of WSS is as follows (19):


F=μ⁢A⁢([v+dv]-v)/dy=μ⁢Adv/dy

WSS=F/A=μ⁢dv/dy

$$\mu \;:\text{ blood viscosity coefficient}=4.0\times 10^{-3}\;\left(N\;\times \;s\;\times \;m^{-2}\right)$$

In the Equation, the flow velocity of a certain layer is v (m/s), the flow velocity of an adjacent layer is v + dv, the dv is the velocity difference, and the vertical distance between two flow layers is dy (m). If the vertical distance is very close, then the velocity difference between flow layers is infinitely close to linear to form a velocity gradient. Thus, the dv/dy represents the velocity gradient, F (N) indicates inner friction between fluid layers, and A (m^2^) is the contact area. WSS (N/m^2^), which is a vector with size and direction, is equal to the inner friction per unit area or the product of blood viscosity (μ) and dv/dy. μ is blood viscosity assumed to be 4.0 × 10^–3^ (N × s × m^–2^) in patients with normal hematocrit (Figure 3B). The direction of WSS is opposite to the direction of blood flow (20, 21). In the following, we used the absolute value to represent WSS.

### Reproducibility

Intraobserver variation in P1 inferoseptal segment WSS and P2 inferoseptal segment WSS were assessed in subjects, with the second analysis performed 2 weeks after the first analysis. Interobserver variation was assessed by comparing the two quantitative measurements made by the first echocardiographer with those of the blinded second echocardiographer. Reproducibility was assessed by Bland-Altman analysis.

### Statistical analysis

Statistical analysis was performed using the SPSS statistic software (IBM SPSS, version 22.0, Chicago, IL, USA). Continuous data with normal distributions were expressed as the mean ± standard deviation (SD), and those with skewed distributions were presented as medians (25th and 75th percentiles). The various trends of LV WSS among four phases were evaluated by one-way repeated measures analysis of variance (ANOVA). A comparison of LV WSS among different age groups was performed using the Kolmogorov–Smirnov test. Comparisons between men and females were performed by the independent-samples *t*-tests. A *P* < 0.05 was considered statistically significant.

## Results

### Clinical and conventional echocardiographic parameters

A total of 375 healthy subjects satisfying the inclusion criteria were originally recruited for this study. However, four participants were excluded because of the poor image quality. Finally, 371 subjects were included in the analysis. The clinical data and echocardiographic measurements of all eligible subjects are listed based on different age groups in Table 1. The peak velocity A of the mitral valve and the Ave E/e′ ratio increased with age, whereas the peak velocity E of the mitral valve, the medial and lateral e′ velocities of the mitral annulus, and the E/A ratio all decreased with age (*P* < 0.05).

**TABLE 1:** Clinical and echocardiographic measurements of the study populations.

	Group I (*N* = 100)	Group II (*N* = 92)	Group III (*N* = 96)	Group IV (*N* = 83)	*P*
Age, years	22.50 ± 3.17	37.59 ± 3.83	50.31 ± 3.88	62.35 ± 3.79	
BMI, kg/m^2^	21.71 ± 3.02	23.11 ± 4.17	23.79 ± 2.69	23.01 ± 2.71	0.042
Heart rate, beats/min	72.00 ± 10.00	76.00 ± 10.00	73.00 ± 9.00	80.00 ± 7.00	<0.001
LVEDD, mm	42.82 ± 4.04	44.70 ± 3.90	43.59 ± 3.85	44.43 ± 4.34	0.004
LVESD, mm	25.17 ± 3.68	24.78 ± 3.54	23.14 ± 4.00	23.62 ± 3.52	<0.001
IVS, mm	8.62 ± 1.96	9.32 ± 1.25	9.34 ± 1.51	8.90 ± 1.81	0.005
LVPW, mm	8.51 ± 1.29	9.16 ± 1.24	9.31 ± 1.29	8.90 ± 1.26	<0.001
LAVI, ml/m^2^	8.63 ± 2.69	10.14 ± 3.01	9.87 ± 3.67	11.32 ± 3.78	<0.001
PAD, mm	19.29 ± 2.73	20.05 ± 2.23	20.04 ± 2.80	20.47 ± 2.45	0.013
LVEF, %	67.37 ± 3.51	67.31 ± 3.99	67.25 ± 3.92	66.98 ± 4.14	0.920
Peak E-wave velocity, cm/s	83.65 ± 17.03	76.37 ± 18.08	69.63 ± 16.99	68.12 ± 15.29	<0.001
Peak A-wave velocity, cm/s	47.56 ± 12.05	54.43 ± 12.84	60.47 ± 15.13	72.66 ± 16.96	<0.001
MV E/A ratio	1.87 ± 0.64	1.44 ± 0.34	1.25 ± 0.72	0.97 ± 0.28	<0.001
Mitral lateral e′, cm/s	18.37 ± 3.89	14.43 ± 3.02	11.68 ± 2.77	10.53 ± 2.29	<0.001
Mitral lateral E/e′	4.73 ± 1.24	5.45 ± 1.40	6.07 ± 1.64	6.64 ± 1.55	<0.001
Mitral septal e′, cm/s	12.62 ± 2.27	10.95 ± 7.80	8.28 ± 1.91	7.58 ± 1.62	<0.001
Mitral septal E/e′	6.54 ± 1.34	7.24 ± 3.12	8.44 ± 2.01	9.09 ± 2.02	<0.001
Mitral ave E/e′	5.63 ± 1.29	6.34 ± 2.26	7.25 ± 1.82	7.86 ± 1.78	<0.001
Tei index	0.35 ± 0.04	0.36 ± 0.05	0.38 ± 0.11	0.35 ± 0.05	0.027
Vp, cm/s	77.59 ± 14.26	76.02 ± 16.12	76.29 ± 15.64	74.03 ± 18.59	0.513
E/Vp	0.11 ± 0.03	0.10 ± 0.03	0.10 ± 0.03	0.10 ± 0.04	0.002

Measurements are shown as means ± SD; BMI, body mass index; LVEDD, left ventricular end-diastolic diameter; LVESD, left ventricular end-systolic diameter; IVS, interventricular septum; LVPW, left posterior ventricular wall; LAVI, left atrial volume index; PAD, pulmonary artery diameter; LVEF, left ventricular ejection fraction; Vp, propagation velocity of early diastolic mitral inflow; and *P* < 0.05: The difference is statistically significant.

### Wall shear stress characteristics in different diastolic phases

**FIGURE 4:**
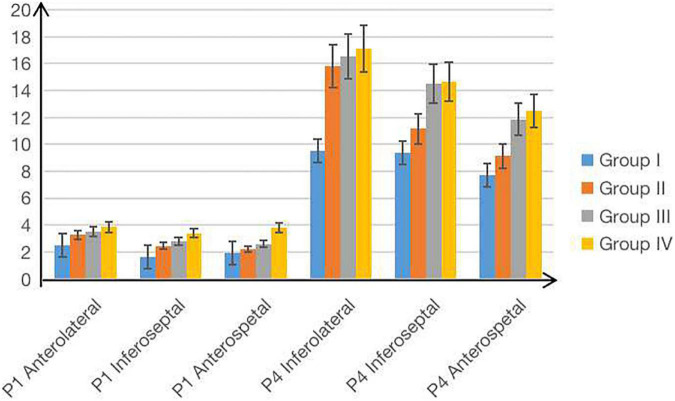
The increasing trend of the LV WSS in four age groups.

Since the plus-minus sign only represents the direction of WSS, we used the absolute value to represent WSS. For each age group, LV WSS showed significant differences in different diastolic phases. WSS demonstrated the increasing-decreasing-increasing trend from P1 to P4 and reached its peak in the rapid filling phase (P2). Moreover, LV WSS had significant phase-related differences in each age group, such as WSS of anterior and inferoseptal segments in each diastolic phase, inferior segment WSS in P2, P3, and P4, WSS of anterolateral and inferolateral segments in P2 and P4, and anteroseptal segment WSS in P3 and P4. Particularly, WSS of anterolateral, inferoseptal, and anteroseptal segments in P1 and WSS of inferolateral, inferoseptal, and anteroseptal segments in P4 all showed an increasing trend with age among four groups (*P* < 0.05, Table 2 and Figure 4). Women generally had greater diastolic WSS than men (*P* < 0.05, Table 3). These WSS sex differences were found in inferolateral and anteroseptal segments in P1, anterior and anterolateral segments in P2, and inferoseptal segments in P3.

**TABLE 2:** The LV WSS values in the diastolic period stratified by different age groups.

WSS values (N/m^2^)		Group I (*N* = 100)	Group II (*N* = 92)	Group III (*N* = 96)	Group IV (*N* = 83)	*P*
P1	Anterior	2.80 ± 1.78	5.29 ± 3.17	2.07 ± 1.55	4.85 ± 2.03	<0.001
	Anterolateral	2.51 ± 0.87	3.28 ± 1.98	3.52 ± 2.62	3.85 ± 2.35	<0.001
	Inferolateral	3.80 ± 2.55	4.61 ± 3.24	3.33 ± 2.49	4.17 ± 2.29	0.028
	Inferior	2.59 ± 1.48	3.62 ± 2.73	4.13 ± 2.55	3.16 ± 1.90	<0.001
	Inferoseptal	1.60 ± 1.34	2.46 ± 1.37	2.80 ± 1.89	3.40 ± 2.22	<0.001
	Anterospetal	1.89 ± 1.42	2.19 ± 1.45	2.59 ± 1.90	3.79 ± 1.98	<0.001
P2	Anterior	33.64 ± 19.60	30.74 ± 22.60	46.36 ± 31.64	53.90 ± 35.74	<0.001
	Anterolateral	25.82 ± 16.71	11.09 ± 5.94	28.50 ± 17.09	36.81 ± 25.45	<0.001
	Inferolateral	16.42 ± 12.81	21.76 ± 15.24	35.72 ± 20.54	25.15 ± 17.40	<0.001
	Inferior	25.62 ± 14.47	42.90 ± 29.86	26.48 ± 14.64	23.57 ± 16.11	<0.001
	Inferoseptal	31.44 ± 22.39	24.95 ± 17.78	22.62 ± 10.80	22.83 ± 11.78	0.006
	Anterospetal	31.35 ± 20.69	33.06 ± 28.17	34.47 ± 24.80	31.80 ± 22.62	0.963
P3	Anterior	2.25 ± 1.13	2.08 ± 0.88	9.44 ± 4.99	2.09 ± 1.12	<0.001
	Anterolateral	2.95 ± 2.20	0.44 ± 2.28	1.77 ± 1.20	1.81 ± 0.87	<0.001
	Inferolateral	3.20 ± 2.10	4.22 ± 2.59	2.09 ± 1.26	2.46 ± 1.69	<0.001
	Inferior	2.39 ± 1.54	2.51 ± 1.82	0.81 ± 0.58	2.16 ± 1.50	<0.001
	Inferoseptal	1.75 ± 1.60	2.13 ± 1.16	1.12 ± 0.94	2.01 ± 1.49	<0.001
	Anterospetal	4.71 ± 3.31	1.39 ± 0.99	1.39 ± 0.91	1.96 ± 1.25	<0.001
P4	Anterior	4.49 ± 2.87	10.77 ± 5.52	25.81 ± 16.81	25.42 ± 14.99	<0.001
	Anterolateral	9.44 ± 5.41	10.53 ± 6.93	24.63 ± 18.25	19.80 ± 10.75	<0.001
	Inferolateral	9.48 ± 4.95	15.80 ± 8.60	16.54 ± 8.59	17.10 ± 9.80	<0.001
	Inferior	8.98 ± 3.56	14.94 ± 6.30	22.83 ± 12.47	21.89 ± 19.27	<0.001
	Inferoseptal	9.39 ± 4.54	11.17 ± 5.46	14.53 ± 4.90	14.66 ± 5.61	<0.001
	Anterospetal	7.73 ± 4.45	9.14 ± 3.52	11.85 ± 5.58	12.48 ± 8.26	<0.001

Measurements are shown as means ± SD; WSS, wall shear stress; *P* < 0.05: The difference is statistically significant.

**TABLE 3:** Differences in LV WSS values between men and women at diastolic period.

WSS values (N/m^2^)		Men (*N* = 146)	Women (*N* = 225)	*P*
P1	Anterior	3.56 (3.93)	3.09 (3.46)	0.257
	Anterolateral	2.95 (2.62)	2.85 (2.54)	0.671
	Inferolateral	4.21 (4.20)	3.56 (3.44)	0.128
	Inferior	3.32 (3.64)	3.15 (2.96)	0.838
	Inferoseptal	2.11 (2.59)	2.32 (2.33)	0.839
	Anterospetal	2.32 (2.56)	2.38 (2.65)	0.686
P2	Anterior	30.21 (33.86)	38.93 (41.25)	0.021
	Anterolateral	17.03 (19.86)	23.08 (28.27)	<0.001
	Inferolateral	20.48 (20.82)	21.79 (22.47)	0.819
	Inferior	23.28 (25.13)	26.73 (21.84)	0.820
	Inferoseptal	22.07 (18.12)	24.75 (19.43)	0.325
	Anterospetal	25.77 (32.48)	31.58 (30.87)	0.142
P3	Anterior	2.33 (1.96)	2.44 (2.38)	0.679
	Anterolateral	1.68 (2.09)	1.92 (2.13)	0.059
	Inferolateral	3.11 (3.62)	2.77 (2.31)	0.114
	Inferior	0.70 (2.86)	1.09 (2.54)	0.060
	Inferoseptal	1.34 (1.67)	1.75 (1.94)	0.025
	Anterospetal	1.95 (2.39)	1.82 (2.44)	0.657
P4	Anterior	9.07 (13.68)	11.58 (21.78)	0.199
	Anterolateral	11.17 (11.83)	13.76 (15.40)	0.019
	Inferolateral	13.18 (10.33)	12.08 (12.07)	0.116
	Inferior	13.79 (11.43)	12.93 (16.61)	0.281
	Inferoseptal	12.98 (7.06)	11.39 (7.54)	0.061
	Anterospetal	10.38 (5.81)	8.58 (7.49)	0.008

Measurements are shown as medians (25th, 75th percentiles); *P* < 0.05: The difference is statistically significant.

### Interobserver and intraobserver reproducibility and repeatability

**FIGURE 5:**
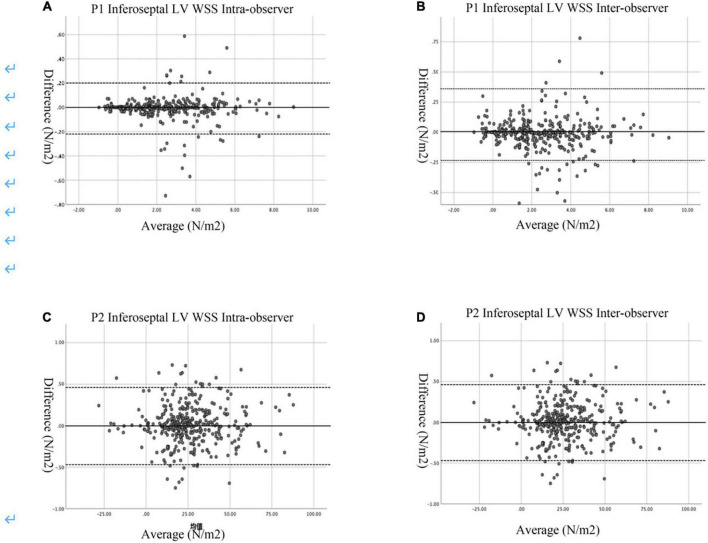
Bland–Altman plots of intra- and inter-observer variability. **(A)** Intra-observer variability in the P1 Inferoseptal LV WSS. **(B)** Inter-observer variability in the P1 Inferoseptal LV WSS. **(C)** Intar-observer variability for the P2 Inferoseptal LV WSS. **(D)** Inter-observer variability for the P2 Inferoseptal LV WSS. The mean values of pairs of measurements are plotted against the difference between the measurements. The black continuous line represents the arithmetic mean and the black dotted lines represent 95% limits of agreement.

The Bland–Altman analyses indicated that the limits of agreement for P1 inferoseptal segment LV WSS (intraobserver: −1.88 to 3.23% and interobserver: −1.08 to 4.31%) and P2 inferoseptal segment LV WSS (intraobserver: −3.77 to 3.23% and interobserver: −3.77 to 1.88%; Figure 5) were good.

## Discussion

The temporal and spatial distribution of blood flow velocity contributes to providing the diagnostic and prognostic information on CVDs (22). Because the inner membrane of the ventricular wall is not smooth, the blood flow pattern is disturbed. Viscous inner friction is produced when the near-wall blood flows through the wall due to the viscosity of the blood. Thus, it is essential to characterize and quantify the flow-wall interaction for evaluating the LV structure and function. WSS is the inner friction per unit area, also representing a vector field opposite to the direction of the blood flow. WSS can quantify the interaction of the intraventricular flow vector and the wall (12), thus, reflecting the changes in cardiac structure and function. Although WSS can be measured by magnetic resonance imaging (MRI) (23, 24), the clinical application of MRI is limited for its long examination duration, high costs, and low temporal resolution. Echocardiographic VFM technology is a novel visualization approach based on the method proposed by Garcia et al. (20). It could serve as an ideal tool to visualize the intraventricular flow vector, which may accurately evaluate the local and global hemodynamics during the left ventricular diastolic period, and reflect the corresponding left ventricular diastolic function (25). In the VFM technology, Gaussian filtering is employed to remove noise and non-smooth factors to smooth the blood flow without producing many errors (20). However, to date, almost no research on the LV WSS has been conducted; hence, the purpose of our study was to investigate the changing trend of the LV WSS in normal people to prepare for future research on patients with certain diseases. In general, diastolic dysfunction often occurs prior to systolic dysfunction. Therefore, early detection of diastolic LV WSS is very important.

In the period before the mitral valve opening and after aortic valve closure, the flow velocity in the LV cavity is lower than that in other diastolic phases. Then, it dramatically increases with the flow rapidly passing from the left atrium into the LV (3). The velocity gradually decreases when it turns to the outflow tract after reaching the apex. However, with the left atrium contracting, the flow enters the LV again, resulting in increased flow velocity (3, 22, 26). In our study, LV WSS coincided with the increasing-decreasing-increasing trend during P1–P4 because the change in WSS is related to the blood flow velocity during the diastolic period. According to the Newton inner friction equation, WSS increases with the blood flow velocity (21).

The prevalence of triglycerides and low-density lipoprotein cholesterol significantly increases with age (27). Studies have found that elevated levels of plasma triglycerides and low-density lipoprotein cholesterol were associated with the increase in blood viscosity (28). WSS in certain segments gradually increases with age, probably because WSS is directly proportional to blood viscosity. Fleg and Strait (29) have demonstrated a shortening of the LV along its long axis and a shift from an elongated prolate ellipsoid geometry to a more spherical LV with age. As a result, a more spherical ventricle was subject to higher wall stress. In our study, women generally had greater diastolic WSS than men. One plausible reason may be the higher level of testosterone in men, which may shorten the QT interval and result in a shorter action potential duration (30). In addition, compared with men, women might have a stronger cardiac response to demand. Thus, gender could present as a factor when evaluating the WSS value (31). The above-mentioned might be the reason why women generally had greater diastolic WSS than men.

Previous researches have also shown that WSS is directly related to vascular function. High WSS could regulate the inner diameter of a blood vessel and inhibit the increase in blood vascular wall, thrombosis, and inflammation. However, lower WSS was known to express an atherogenic endothelial gene profile, as observed in the carotid arteries in subjects with risk factors for atherosclerosis (32). Additionally, WSS also plays a vital role in hypertension patients, which is achieved by directly or indirectly releasing bioactive molecules (33, 34). Most of the previous studies were focused on vascular, meanwhile, abnormal WSS could also triggers a series of pathological changes in cardiac endothelial cells, which in turn affect cardiac function. Our study explored the spatial and temporal distribution patterns of WSS during different cardiac cycles in normal subject, which is also a preparation for the future study of left ventricular dysfunction. Ji et al. (11) have reported that the LV vortex and WSS were evaluated in patients with hypertrophic cardiomyopathy. Their results have shown that, compared with the control group, hypertrophic cardiomyopathy patients showed increased WSS at the peak of the LV rapid ejection period and the atrial contraction period and decreased WSS at the end of early diastole. The above study indicated that WSS could be a parameter to assess left ventricular dysfunction in patients with hypertrophic cardiomyopathy (32).

Certain limitations should also be noted in this study. First, the VFM technology can only evaluate the flow field vector in the 2D echocardiography, and there is still a certain distance from the accurate measurement of the LV complex three-dimensional flow field vector. Second, the current study is a preliminary study based on a small sampling, while the changes in WSS must be verified in a larger and more diverse study. Third, the blood cell contents are different among various individuals, also resulting in different blood viscosity coefficients (7). Thus, it requires further research.

To summarize, LV WSS in normal subjects showed certain changes during the diastolic period in our current cohort, which coincided with the flow changes in LV and might provide valuable information for the assessment of the LV diastolic function. Further study needs to be performed to verify its clinical value in evaluating the diastolic function for both healthy individuals and patients with certain diseases.

## Data availability statement

The original contributions presented in this study are included in the article/Supplementary material, further inquiries can be directed to the corresponding author/s.

## Ethics statement

The studies involving human participants were reviewed and approved by the Ethical Committee of The Second Affiliated Hospital of Harbin Medical University. The patients/participants provided their written informed consent to participate in this study.

## Author contributions

LD, HL, and XX contributed to the writing of the manuscript and data analysis. LD, MR, and WY contributed to the revision of the manuscript and the design of statistical charts. WB and DS contributed to the revision of the manuscript. JT contributed to the entire experimental process. All authors contributed to the article and approved the submitted version.
